# CD44 in Differentiated Embryonic Stem Cells: Surface Expression and Transcripts Encoding Multiple Variants

**DOI:** 10.1155/1994/25484

**Published:** 1994

**Authors:** Hélène Haegel, Andrée Dierich, Rhodri Ceredig

**Affiliations:** INSERM U184 CNRS LGME Institut de Chimie Biologique Faculté de Médecine, 11 rue Humann Strasbourg Cedex 67085 France

**Keywords:** CD44, embryonic stem cells, alternative splicing, adhesion molecules

## Abstract

Expression of the surface-adhesion molecule CD44 was investigated during the *in vitro* differentiation of the embryonic stem (ES) cell line D3. By immunofluorescence analysis, totipotent, undifferentiated ES cells did not show surface expression of CD44, although two transcripts of approximately 1.6 and 3.3 kb were detected on Northern blots. Following 1 week of differentiation in either suspension or substrate-attached cultures, CD44 appeared on the surface of some D3 cells, and synthesis of an additional 4.5 kb mRNA species was detected on Northern blots. At this stage, at least three distinct transcripts encoding CD44 variants were induced within the cultures, resulting from alternative splicing of additional exons in the variable domains of CD44. From PCR analysis, they all appeared to contain the variable exon v10, and two of them in addition contained v6. Taken together, these results suggest that CD44 may play a role in cell migration and adhesion in the early development of the mouse embryo.


*Developmental Immunology*, 1994, Vol 3, pp. 239–246
Reprints available directly from the publisher
Photocopying permitted by license only

© 1994 Harwood Academic Publishers GmbH
Printed in Singapore

### CD44 in Differentiated Embryonic Stem Cells: Surface Expression and Transcripts Encoding Multiple Variants

HÉLÈNE HAEGEL,† ANDRÉE DIERICH,† and RHODRI CEREDIG*†

*INSERM U184, CNRS LGME, Institut de Chimie Biologique, Faculté de Médecine, 11 rue Humann, 67085 Strasbourg Cedex, France*

Expression of the surface-adhesion molecule CD44 was investigated during the *in vitro* differentiation of the embryonic stem (ES) cell line D3. By immunofluorescence analysis, totipotent, undifferentiated ES cells did not show surface expression of CD44, although two transcripts of approximately 1.6 and 3.3 kb were detected on Northern blots. Following 1 week of differentiation in either suspension or substrate-attached cultures, CD44 appeared on the surface of some D3 cells, and synthesis of an additional 4.5 kb mRNA species was detected on Northern blots. At this stage, at least three distinct transcripts encoding CD44 variants were induced within the cultures, resulting from alternative splicing of additional exons in the variable domains of CD44. From PCR analysis, they all appeared to contain the variable exon v10, and two of them in addition contained v6. Taken together, these results suggest that CD44 may play a role in cell migration and adhesion in the early development of the mouse embryo.

KEYWORDS: CD44, embryonic stem cells, alternative splicing, adhesion molecules.

### INTRODUCTION

CD44 is a family of cell-surface glycoproteins, encoded by a single complex gene. In the adult animal, the CD44 antigen is expressed by many cell types: hematolymphoid cells; several types of epithelia; and a variety of mesenchymal tissues, including fibroblasts, smooth muscle cells, and astrocytes (Trowbridge et al., 1982; Flanagan et al., 1989; Picker et al., 1989; Kennel et al., 1993). The extracellular, N-terminal part of the CD44 molecule, whose molecular mass is 85–90 kD, comprises a binding site for hyaluronic acid (HA). Some forms of the CD44 antigen can also be associated with chondroitin sulfate to yield a protein of 180–200 kD. CD44 is implicated in cell–cell and cell–substrate adhesion. It can bind several ligands such as collagen and fibronectin, which are important components of extracellular matrixes (ECM) (Carter and Wagner, 1988; Jalkanen and Jalkanen, 1992). In addition, CD44 was found to be a major receptor for hyaluronate (HA) (Aruffo et al., 1990; Lesley et al., 1990; Miyake et al., 1990b). Many functions have been attributed to CD44 molecules, relating to T-cell and B-cell ontogeny (Hyman et al., 1986; Miyake et al., 1990a), cell activation (Budd et al., 1987; Tabi et al., 1988, Mobley and Dailey, 1992), lymphocyte extravasation across endothelial barriers (reviewed by Berg et al., 1989), degradation of HA (reviewed by Underhill, 1992), and tumor metastasis (Günthert et al., 1991).

In addition to the "standard" form of CD44 (Zhou et al., 1989), an increasing number of variant CD44 molecules have been described that result from the insertion of any of ten additional variant (v) exons, labeled v1 to v10, alternatively spliced in the extracellular/membrane-proximal domain (Hofmann et al., 1991; Jackson et al., 1992; Screaton et al., 1992; Tolg et al., 1993). Among these variants, the p-meta-1 variant (Günthert et al., 1991), containing the variable exons v4 to v7, has been implicated in the metastatic spread of tumors. Interestingly, a close variant containing the unique additional exon v6 is induced upon lymphocyte activation *in vivo* and appears to function as a homing receptor for lymphocyte entry into lymph nodes (Arch et al., 1992). This diversity in form and function suggests that surface expression of CD44 variants must be finely regulated not only in adult tissues, but also during development.

*Corresponding author.

The early events of embryogenesis can be mimicked *in vitro* by inducing the differentiation of the embryonic stem cell (ES) line D3, derived from a mouse blastocyst (Doetschman et al., 1985, 1987). This cell line has the potential to participate in the formation of all three embryonic germ–layer tissues (ectoderm, mesoderm, and endoderm) when reinjected into the cavity of a mouse blastocyst before reimplantation *in vivo*. ES/D3 cells have been formerly used for differentiation studies *in vitro*, where they can spontaneously give rise to a variety of cell types, in the absence of leukemia inhibitory factor (LIF) (Martin, 1981; Doestschman et al., 1985, 1987). Two culture systems were used. The first is a suspension culture system in which ESD3 cells are able to form highly organized cystic embryoid body structures, which are analogous to postimplantation embryos and contain derivatives of all three germ layers. The second is a monolayer culture on gelatin-coated dishes in the absence of LIF. In such cultures, ES cells grow as aggregates.

We have used both culture methods to examine the expression of CD44 following differentiation *in vitro*. Totipotent ESD3 cells do not express surface CD44, although transcripts hybridizing with the CD44 cDNA are present. By PCR analysis, these transcripts do not appear to encode the "standard" form of the molecule. Surface expression of CD44 can be detected after 7 days in both differentiation culture conditions. At this stage, the mRNA pattern of CD44 has been considerably modified: 4.5-kb band, which is predominant in cells such as thymocytes, a T-cell hybridoma, and astrocytes (Haegel and Ceredig, 1991; Haegel et al., 1993), appeared on Northern blots, and PCR analysis showed the presence of "standard" CD44 transcripts. Moreover, at least three distinct transcripts coding for variant CD44 molecules were detected. All the PCR-identified transcripts appeared to contain the variable exon v10, and at least two of them contained v6. The possible implications of these results regarding the importance of CD44 during embryonic, including hemopoietic, development are discussed.

### MATERIALS AND METHODS

### Culture and Differentiation of ES Cell Line D3

The ES cell line D3 (gift of R. Kemler, Freiburg) was established from a 129/Sv mouse blastocyst. ESD3 cells were first propagated on feeder layers of embryonic fibroblasts inactivated by mitomycin-C (Doetschman et al., 1985). Then they were adapted in our laboratory in culture without feeders on gelatin-coated dishes. To maintain their undifferentiated state, they were grown in the presence of exogenous LIF (1000 U/ml) in high-glucose DMEM medium (Sigma) supplemented with 2 mM L-glutamine, 1 mM sodium pyruvate, 0.1 mM $\beta$-mercato-ethanol, 50 $\mu$g/ml gentamycin, and 15% fetal calf serum (Gibco). D3 cells cultured in DMEM+LIF for less than twenty-five passages were used in this study.

Differentiation was induced by removing LIF from the medium, and culturing the cells either in suspension using hydrophobic Petric dishes (Sterilin, Staffs, UK) or as substrate-attached cultures in 0.1% gelatin-treated dishes. Cells were harvested at the indicated times and used for RNA extraction and immunofluorescence. In the case of adherent cells, cultures were pretreated for 3 min at 37°C with 0.04% trypsin. Previous studies had shown that this treatment was insufficient to cleave surface CD44 molecules.

### FCM Analysis

Cultures of undifferentiated or differentiated cells were treated for 30 min at 37°C with 1 mM EDTA in PBS followed by gentle pipetting. The resultant cell suspension was filtered through nylon mesh to remove remaining cell clumps and then centrifuged over Hypaque-Ficoll to remove cell debris. The resultant cell suspension was washed twice in DMEM prior to immunofluorescent staining. Cells ($3\times10^5$) in wells of round-bottomed microtitre plates were stained for 30 min at 4°C with saturating concentrations of rat monoclonal antibodies (mAbs) to either CD44 (IM7, $IgG_{2b}$), CD25 (PC61, $IgG_1$) or CD4 (H-129–19.6, $IgG_{2b}$). Following two washes in DMEM without serum, bound mAbs were revealed with mouse-absorbed FITC-labeled sheep anti-rat Ig (Silenus). Prior to addition to labeled ES cells, this second-step reagent was absorbed with unlabeled ES cells and centrifuged at 10,000 g in a microfuge, in order to reduce any nonspecific staining. FCM was carried out with a Coulter Elite flow cytometer and viable cells identified by a combination of narrow angle forward- and side-scatter signals.

### RNA Preparation and Northern Blot Analysis

Total RNA was extracted by centrifugation on a 5.7 M CsCl cushion as described (Maniatis et al., 1982), and separated according to size on a 1% agarose formaldehyde gel that was blotted onto a Hybond-N nylon filter (Amersham, les Ulis, France). The amount and quality of the loaded material was checked by methylene blue staining of the filters (Maniatis et al., 1982). Probes were obtained by random priming of the CD44 (Pgp-1) cDNA 1.3-kb EcoR1 fragment from plasmid Prk-5 (Zhou et al., 1989). Following hybridization, filters were washed in 0.1×SSC, 0.1% SDS at 55°C, and exposed onto Kodak X-OMAT films (Rochester, NY) for 1 day to 2 weeks.

### PCR Amplification of CD44 Variant Region

We used oligonucleotides hybridizing to the standard CD44 region in positions 5′ and 3′ to the variable domains (see Fig. 3B: oligos A and D hybridize in the "standard" CD44 exons s5 and s7), and in exons v6 and v7 of the variable domain (Tolg et al., 1993). These oligos were a kind gift from Prof. Peter Herrlich (KFZ Karlsruhe). Approximately 1 microgram of total RNA samples were reverse transcribed using 20 μg/ml oligo dT primer in 50 mM Tris-HCl, 20 mM KCl, 10 mM $MgCl_2$, 5 mM DTT, and 1 mM of each dNTP. AMV reverse transcriptase (9 U) and ribonuclease inhibitor (6 U) (Amersham) were added in a 20-μl volume, and reverse transcription was achieved after 40 min at 42°C. The reaction was stopped by adding 80 μl $H_2O$, and 2 μl of this cDNA preparation was submitted to PCR amplification.

The amplification was carried out in a buffer containing 10 mM Tris-HCl, 50 mM KCl, 2 mM $MgCl_2$, 1% gelatin, and 200 μM of each dNTP. 1 μM of each primer pair and 0.5 unit of TAQ polymerase (Perkin Elmer Cetus) were added in a final volume of 20 μl. Samples were denatured 7 min at 94°C, followed by thirty-five cycles of 30 sec at 92°C, 20 sec at 60°C, and 1 min at 72°C, with a 2 sec extension at each cycle, and a final 10 min elongation at 72°C. PCR products were run on a 2% agarose gel, blotted onto Hybond-$H^+$ (Amersham), and hybridized to CD44 variant region cDNA probes (kind gifts of P. Herrlich) corresponding to exons v6–7 and v10, obtained by random priming. Filters were exposed to Kodak X-OMAT filters for intervals of 1 to 3 days.

### RESULTS

### Totipotent ESD3 Cells Do Not Express Surface CD44 but CD44 Transcripts Are Present

We have examined CD44 transcripts in undifferentiated ESD3 cells (Fig. 1, right panel). The amount of RNA loaded on Northern blots was checked by methylene blue staining of the filters, and ribosomal RNA bands were used as size markers (Fig. 1, lower panel). Two bands were shown to hybridize to a CD44 cDNA probe (Zhou et al., 1989). Their sizes were estimated to be 1.6 and 3.3 kb. They were compared to the CD44 mRNA pattern of a T-cell hybridoma, H11.1 (Fig. 1, left panel), known to express a high level of CD44 protein on the cell surface and to contain transcripts of 1.6, 3.5, and 4.5 kb (Haegel and Ceredig, 1991). The difference in size between CD44 mRNA has been proposed to result from distinct polyadenylation sites or 3′UTR length (Schtivelman and Bishop, 1991). The pattern of CD44 transcripts in ES and H11.1 cells was

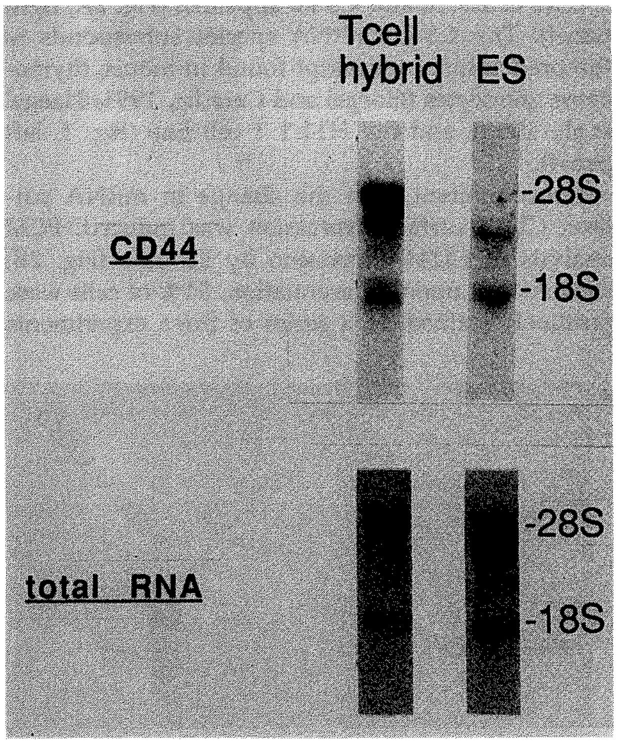

FIGURE 1. CD44 mRNAs in undifferentiated ES/D3 cells (right) compared to the H11.1 hybridoma T cells (left). Northern blot was hybridized to a standard CD44 cDNA probe (upper panel), after staining of total RNA with methylene blue (lower panel). The position of the ribosomal RNA bands of 28S (4.8 kb) and 18S (1.8 kb) is indicated.

strikingly different with only the 1.6-kb transcript being found in both ES and H11.1 cells (Fig. 1).

### *In Vitro* ES Differentiation Induces CD44 Transcripts and Surface Expression in Individual Cells

Two types of culture conditions were used in which ESD3 cells were allowed to differentiate spontaneously in the absence of LIF: either in substrate-attached conditions (on gelatin-coated dishes) or in suspension. We isolated total RNA from cells that had been left to differentiate for varying lengths of time. Already after 1 week and under both culture conditions, the pattern of CD44 mRNAs had been considerably modified (Fig. 2A). Note that compared to the D3 cells used in Fig. 1, those in Fig. 2A (left panel) presented a faint additional mRNA band slightly smaller than the 1.6 kb species. This difference may correspond to the later passage number of cells used in Fig. 2A. Upon differentiation, not only did the bands corresponding to the 3.3- and 1.6-kb mRNAs become more intense, but new mRNA species of 2.5, 3, and 4.5 kb appeared (Fig. 2A, right panel). The 4.5-kb mRNA species corresponds to the predominant transcript found in mouse thymocytes, astrocytes (Haegel and Ceredig, 1991; Haegel et al., 1993), and the H11.1 T-cell line (Fig. 1, left panel).

Concommitant with the change in mRNA pattern, CD44 surface expression was induced. FCM analysis of CD44 expression by ES cells (Fig. 2B) showed that upon differentiation, 31% of cells were positively stained. In a series of three experiments with ES cells differentiated for 3 weeks in either culture conditions, a mean of 25% of cells were positively stained. Shown in Fig. 2B are the staining profiles with an irrelevant rat mAb to CD 25. Similar low-level staining was obtained with a rat Ig subclass-matched ($IgG_{2b}$) anit-CD4 mAb H-129-19.6 (data not shown).

### Induction of Variant CD44 mRNAs Upon ES Cell Differentiation

We investigated the presence of transcripts encoding CD44 variants in ES cell cultures. Northern blots of total RNA from ESD3 or differentiated cultures were screened using a probe corresponding to exons v4 to v10. However, we could not obtain detectable signals using this technique. Therefore, a PCR strategy was chosen (see scheme in Fig. 3, lower panel). Combinations of four oligonucleotides were used in experiments where reverse-transcribed total RNA was subjected to PCR. Oligos A and D (lanes 3 and 6 in Fig. 3) should be able to amplify the whole variable domain, from the 5′ to the 3′ constant region. Oligos B and D (lanes 1 and 4 in Fig. 3) amplify the region located between v6 and 3′ constant region, whereas A and C (lanes 2 and 5 in Fig. 3) amplify from the 5′ constant region up to and including v7. The PCR products were submitted to electrophoresis on a 2% agarose gel. Given the very low amount of PCR-amplified material detected by ethidium bromide staining (Fig. 3, lower panel), gels were Southern blotted with probes corresponding to either the v6–v7 (Fig. 3, upper panel) or the v10 exon (lower panel).

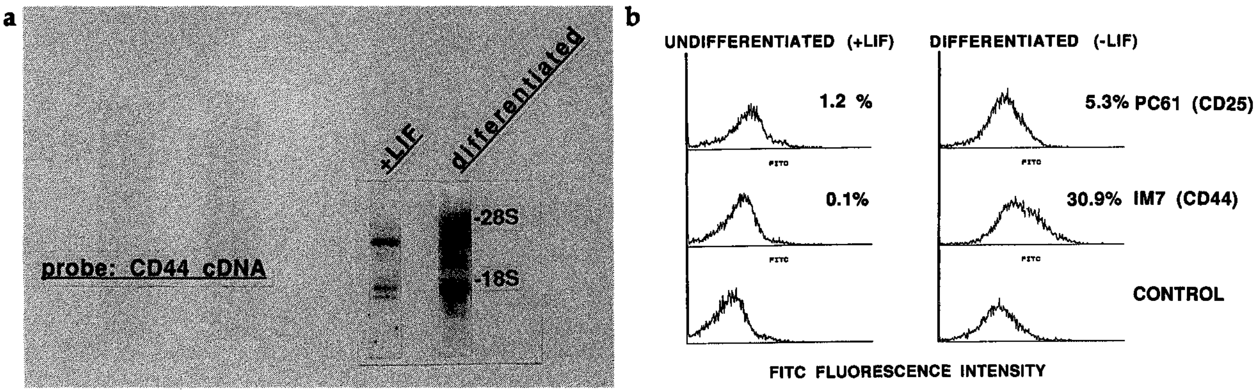

FIGURE 2. *In vitro* differentiation of ES/D3 cells induces CD44 mRNAs and surface expression. (a) Northern blot on total RNA of undifferentiated ES cells cultured in LIF (left), and ES cells differentiated for 1 week in substrate-attached cultures (right), hybridized to the standard CD44 cDNA probe. (b) FCM analysis of CD44 expression. Shown are fluorescence histograms of undifferentiated (left panels) or differentiated (right panels) ES cells stained with (from bottom to top) the second step reagent alone (Control), anti-CD44 (middle histograms), or anti-CD25 (upper histograms). The figures in each panel show the percentage cells staining above the control.

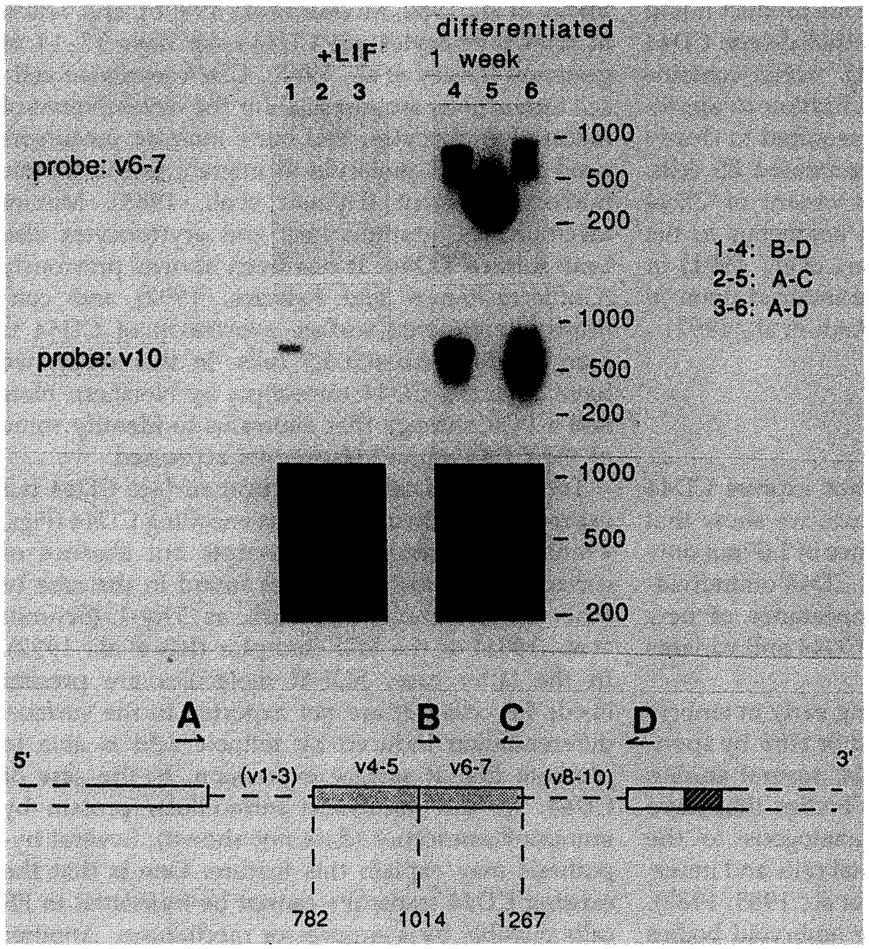

FIGURE 3. PCR amplification of variant CD44 transcripts in undifferentiated ES/D3 cells and 1-week-differentiated cultures. At the bottom of the figure is shown a schematic structure of the p-meta-1 variant (Günthert et al., 1991). Standard CD44 sequences (open bars), exons from the variant region (shaded bars), and the transmembrane region (hatched bar) are shown. Localization of the additional exons (dashed lines) identified in larger CD44 variants is according to Arch and colleagues (1992). Oligonucleotides A, B, C, and D hybridize to positions 653–680, 1039–1070, 1146–1173, and 1328–1356, respectively. At the top, cDNA from undifferentiated ES/D3 cells or 1-week-differentiated cultures were amplified using the primer pairs indicated. The PCR products separated in 2% agarose gel, stained with ethidum bromide (bottom panel) were Southern blotted and hybridized to cDNA probes corresponding either to variable exons v6–v7 (upper panel) or to exon v10 (middle panel).

In undifferentiated ESD3 cells, oligos A and C or D did not amplify a detectable transcript. However, oligos B and D amplified one band containing exon v10 (lane 1 in Fig. 3). This transcript also contained v6, because the fragment was amplified using oligo B in this exon, but the corresponding signal was very faint by hybridization to v6–v7. From the size of this PCR product (approx. 750 bp), it could be assumed that all additional exons from v6 to v10 were present in this transcript.

In ES cells that had been left to differentiate for 1 week either in suspension or in substrate-attached conditions, PCR amplification with the external oligos A and D and ethidium bromide staining of the gel revealed a band of 290 bp corresponding to the "standard" form of CD44 (Fig. 3, lower right panel). As noted before, this 290-bp band was absent in undifferentiated ES cells (Fig. 3, lower left panel), suggesting that the sequence corresponding to oligo A (located in the 5′ constant exon 5) may be missing in the CD44 transcripts of these cells.

Upon differentiation, a dramatic induction of at least three distinct variant-encoding CD44 transcripts was detected (Fig. 3, upper panels, lanes 4–6). All additional transcripts contained the variable exon v10. At least two contained exon v6 (Fig. 3, upper panel, lane 4), and at least one of them contained exon v7 (Fig. 3, upper panel, lane 5). By analyzing the sizes of these transcripts, it appears that (1) one of the variant CD44 transcripts contains all additional exons from v6 to v10; (2) another variant CD44 mRNA contains exons v6 and v10 and one unique additional exon in between (v7, v8, or v9); and (3) the smallest variant identified is composed of only two additional exons, either v6 or v7 in combination with v10.

In addition, a 600-bp band hybridizing with v10 but not v6 appears to be induced upon differentiation (Fig. 3, upper right, lane 6). This product might result from the presence of an epithelial-type CD44 variant (Stamenkovic et al., 1991), which contains exons v8 to v10. However, hybridization to appropriate oligonucleotides would be required to clearly characterize this variant. In differentiated ES cells, we have not detected either the variant of CD44 containing exon v6 but not v10, analogous to the v4–v7 variant p-meta-1 (Günthert et al., 1991) or the v6-only variant, known to be induced upon *in vivo* activation of lymphocytes (Arch et al., 1992).

### DISCUSSION

While totipotent ESD3 cells do not express CD44 adhesion molecules on their surface, we show that *in vitro* differentiation in the absence of LIF not only results in the induction of surface CD44 on individual cells, but also allows the appearance of new transcripts coding for "standard" CD44 and variants containing exon v10.

The sequence of events occurring early in embryonic development can be followed *in vitro* by spontaneous differentiation of ES cells; several studies show that the simple embryoid bodies that have developed within 6 days are analogous to the 4.5-day blastocyst, with endodermal cells and inner-cell mass stem cells (Doetschman et al., 1985, 1987). From day 6 to day 8 *in vitro*, the embryoid bodies become complex as ectoderm like cells develop underneath basal lamina. This stage is related to day 5.5 of embryonic development. After 8 days in culture, complex embryoid bodies become cystic (CEBs) and appear analogous to the visceral yolk sac (days 9–10). CEBs contain cell types of mesodermal origin: a variety of hematopoietic cells such as erythroid cells, granulocytes, macrophages, pro-B and pro-T lymphoid progenitors develop in blood islands (Doetschman et al., 1985; Schmitt et al., 1991; Wiles and Keller, 1991; Chen, 1992; Guttierez-Ramos and Palacios, 1992; Keller et al., 1993). Myocardium, endocardium, and neural cell types (Doetschman et al., 1987) can also be obtained by *in vitro* culture of ESD3 cells in the absence of LIF. Many attempts are being made toward monitoring ES cultures in order to induce specific differentiation pathways.

CD44 expression has been extensively characterized in the adult mouse and particularly along hematopoietic cell lineages. CD44 expression appears early during T-cell and B-cell ontogeny (Trowbridge et al., 1982; Miyake et al., 1990b), and >80% of fetal thymocytes are CD44+ by days 13–14 of gestation (Lesley et al., 1985). CD44-mediated cell–cell interactions are essential for the development of mature lymphocytes, and bone marrow precursors need the CD44 molecule to migrate to the thymic microenvironment (Hyman et al., 1986). Mature macrophages, granulocytes, and erythrocytes also bear surface CD44. It has been shown previously (Guttierez-Ramos and Palacios, 1992) and confirmed herein that surface expression of CD44 is seen on differentiated ES cells. In this study, we have analyzed CD44 transcripts by Northern blots and a PCR strategy that allows us to identify some of the CD44 variant transcripts expressed.

Totipotent ES cells do not bear surface CD44 but express at least two transcripts encoding CD44 (Figs. 1 and 2). Presence of transcripts but absence of surface expression have been found in the case of other surface molecules, such as Thy-1 (Schmitt et al., 1991) or the EGF-Receptor (Joh et al., 1992). In the latter case, EGF-R molecules are present inside ES cells but are not exported to the surface; differentiation induced by retinoic acid is able to promote EGF-R surface expression. In the case of CD44, we did not detect intracellular protein by immunofluorescence (data not shown). Several hypothesis may explain this feature: One is that the existing CD44 transripts cannot be translated in ES cells possibly by a suppressor mechanism. Another is that CD44 molecules are synthesized but are not exported to the cell surface. Immunofluorescence may not be a sensitive enough technique to detect small numbers of surface molecules. Because by PCR analysis of undifferentiated ES cells, transcripts encoding "standard" CD44 were not amplified, an alternative possibility is that the bands detected on Northern blots may not encode complete "standard" CD44 molecules. A deletion in the standard CD44 sequence 5′ to the variable region is suggested by our PCR results. Indeed, a splice site within the s5 exon of human CD44 was previously reported by Screaton and colleagues (1992). In the mouse, this splice donor site has not been demonstrated but it is possible that a splicing takes place in the s5 exon.

In a previous study, we found a correlation between CD44 surface expression and the presence of the 4.5 kb mRNA band on Northern blots of mouse thymocytes and T-cell hybridoma (Haegel and Ceredig, 1991). Whether the lack of detectable surface CD44 expression on undifferentiated ES

cells is due to the absence of the 4.5 kb transcript cannot be excluded.

In our ES cultures, the induction of three variant CD44 transcripts (Fig. 3) also correlated with the time when CD44+ cells arose in the cultures. Because monoclonal antibodies to variant parts of mouse CD44 molecules were not available, we were unable to determine whether proteins containing these variable exons were effectively expressed on the cell surface. As mentioned previously, about 20% of cells from CEBs cultured for 5 weeks appeared to express CD44 (Fig. 2B and not shown). From the very low amount of variant CD44 transcripts, undetected on Northern blots using oligonucleotide probes to variable exons, it would seem that if efficiently expressed, these variants are quantitatively of minor importance compared to the "standard" CD44. An interesting characteristics of these variants is that they all seem to contain the v10 exon, and at least two of them bear the v6 exon in addition (Fig. 3). The longest appears to be similar to one CD44 variant isolated from the murine carcinoma line KLN205 (He et al., 1992). The variant containing v6 and v10 plus one intermediate exon is close if not identical to one recently described in human carcinomas (Hofmann et al., 1991). In contrast, their structure is clearly distinct from CD44 variants containing v6 but not v10, a variant that can be induced notably by *in vivo* lymphocyte (Arch et al., 1992) or astrocyte (Haegel et al., 1993) activation and expressed on metastatic cell (Günthert et al., 1991). We conclude that these CD44 variants do not appear early during embryonic development. In contrast, variants containing v10 in addition to v6 can be induced very early upon *in vitro* ES development.

Our results led us to speculate on the possible roles of CD44 in early embryonic development. Actin molecules, to which CD44 may be associated (Kalomiris and Bourguignon 1989) have been found concentrated at contact regions between differentiating blastomeres in the mouse preimplantation embryo (Slager et al., 1992). The appearance of CD44 surface expression coincides with the formation of cystic embryoid bodies. At this time (days 8–12 in culture) mesoderm-derived cells have been shown to develop and migrate (Doetschman et al., 1987). In addition, CD44 is known as the main ligand for hyaluronate (HA), which appears to be involved in embryo implantation on mouse endometrium (Brown and Papaioannou, 1992). Moreover, the synthesis of collagen, a ligand for chondroitin-sulfate-associated CD44, is enhanced upon decidualization (Aplin et al., 1988). CD44 expression leading to HA degradation may allow morphological changes in the developing tissues (Underhill, 1992). Finally, when day-8.5 yolk sac cells were cultured in the presence of lymphokines, hemopoietic cells were generated, which by immunofluorescence analysis were CD44+ (Hapel and Ceredig, unpublished observation). Taken together, our results suggest that the CD44 gene is activated early and cD44 molecules may play an important role in early embryonic development, particularly as it relates to hemopoiesis.

*(Received July 13, 1993)*

*(Accepted November 8, 1993)*

### ACKNOWLEDGMENTS

This work was supported by Institutional grants from INSERM, CNRS, CHU Strasbourg, ARC and an ARSEP fellowship to H.H.

### REFERENCES

Aplin J.D., Charlton A.K., and Ayad S. (1988). An immunohistochemical study of human endometrial extracellular matrix during the menstrual cycle and the first trimester of pregnancy. Cell Tissue Res. **253:** 231–240.

Arch R., Wirth K., Hofmann M., Ponta H., Matzku S., Herrlich P., and Zöller M. (1992). Participation in normal immune responses of a metastasis-inducing splice-variant of CD44. Science **257:** 682–685.

Aruffo A., Stamenkovic I., Melnick M., Underhill C., and Seed B. (1990). CD44 is the principal cell surface receptor for hyaluronate. Cell **61:** 1303–1323.

Berg L.E., Goldstein L.A., Jutila M.A., Nakache M., Picker J.L., Streeter P.R., Wu N.W., Zhou D., and Butcher E.C. (1989). Homing receptors and vascular addressins: Cell adhesion molecules that direct lymphocyte traffic. Immunol. Rev. **108:** 5–18.

Brown J.J.G., and Papaioannou V.E. (1992). Distribution of hyaluronan in the mouse endometrium during the preimplantation period of pregnancy. Differentiation **52:** 61–68.

Budd R.C., Cerottini J.C., and MacDonald H.R. (1987). Phenotypic identification of memory cytolytic T lymphocytes in a subset of Lyt-2+ cells. J. Immunol. **238:** 2009–1013.

Carter W.G., and Wayner E.A. (1988). Characterization of the class III collagen receptor, a phosphorylated transmembrane glycoprotein expressed in nucleated human cells. J. Biol. Chem. **263:** 4119–4201.

Chen U. (1992). Differentiation of mouse embryonic stem cells to lympho-hematopoietic lineages *in vitro*. Dev. Immunol. **2:** 29–50.

Doetschman T.C., Eistetter H., Katz M., Schmidt W., and Kemler R. (1985). The *in vitro* development of blastocyst-derived embryonic stem cells: Formation of visceral yolk sac, blood islands and myocardium. J. Embryol. Exp. Morph. **87:** 27–45.

Doetschman T., Gossler A., and Kemler R. (1987). Blastocyst-derived embryonic stem cells as a model for embryogenesis. *In*: Future aspects in human *in vitro* fertilization, Feichtinger W., and Kemeter P., Eds. (Berlin: Springer-Verlag), pp. 187–195.

Flanagan B.F., Dalchau R., Allen A.K., Daar A.S., and Fabre J.W. (1989). Chemical composition and tissue distribution of the human CDw44 antigen. Immunology **67:** 167–175.

Günthert U., Hofmann M., Rudy W., Reber S., Zöller M., Haussmann I., Matzku S., Wenzel A., Ponta H., and Herrlich P. (1991). A new variant of glycoprotine CD44 confers metastatic potential to rat carcinoma cells. Cell **65:** 13–24.

Gutierrez-Ramos J.C., and Palacios R. (1992). *In vitro* differentiation of embryonic stem cells into lymphocyte precursors able to generate T and B lymphocytes *in vivo*. Proc. Natl. Acad. Sci. USA **89:** 9171–9175.

Haegel H., and Ceredig R. (1991). Transcripts encoding mouse CD44 (Pgp-1, Ly-24) antigen: Strain variation and induction by mitogen. Eur. J. Immunol. **21:** 1549–1553.

Haegel H., Tölg C., Hofman M., and Ceredig R. (1993). Activated mouse astrocytes and T cells express similar CD44 variants. Role of CD44 in astrocyte/T cell binding. J. Cell Biol. **122:** 1067–1077.

He Q., Lesley J., Hyman R., Ishihara K., and Kincade P. (1992). Molecular isoforms of murine CD44 and evidence that the membrane-proximal domain is not critical for hyaluronate recognition. J. Cell Biol. **119:** 1711–1719.

Hofmann M., Ruby W., Zöller M., Tölg C., Ponta H., Herrlich P., and Günthert U. (1991). CD44 splice variants confer metastatic behaviour in rats: Homologous sequences are expressed in human tumor cell lines. Cancer Res. **52:** 3486–3490.

Hyman R., Lesley J., Schulte R., and Trotter J. (1986). Progenitor cells in the thymus: Most thymus-homing progenitor cells in the mouse bear Pgp-1 glycoprotein but not IL-2 receptor on their surface. Cell Immunol. **101:** 320–327.

Jackson D.G., Buckley J., and Bell J.I. (1992). Multiple variants of the human lymphocyte homing receptor generated by insertions at a single site in the extracellular domain. J. Biol. Chem. **267:** 4732–4739.

Jalkanen S., and Jalkanen M. (1992). Lymphocyte CD44 binds the COOH-terminal heparin-binding domain of fibronectin. J. Cell Biol. **116:** 817–825.

Joh T., Darland T., Samuels M., Wu J.X., and Adamson E.D. (1992). Regulation of epidermal growth factor gene expression in murine embryonal carcinoma cells. Cell Growth Differ. **3:** 315–325.

Kalomiris E.L., and Bourguignon L.T.W. (1989). Lymphoma protein kinase C is associated with the transmembrane glycoprotein gp85, and may function in gp85-ankyrin binding. J. Biol. Chem. **263:** 4193–4201.

Keller G., Kennedy M., Papayannopoulos T., and Wiles M.V. (1993). Hematopoietic commitment during embryonic stem cell differentiation in culture. Mol. Cell. Biol. **13:** 473–486.

Kennel S.J., Lankford T.K., Foot L.J., Shinpock S.G., and Stringer C. (1993). CD44 expression on murine tissues. J. Cell. Sci **104:** 373–382.

Lesley J., Hyman R., and Schulte R. (1985). Evidence that Pgp-1 glycoprotein is expressed on thymus-homing progenitor cells of the thymus. Cell. Immunol. **91:** 397–403.

Lesley J., Schulte R., and Hyman R. (1990). Binding of HA to lymphoid cell lines is inhibited by monoclonal antibodies against Pgp-1. Exp. Cell. Res. **187:** 224–293.

Maniatis T., Fritsch E.F., and Sambrook J. (1982). Molecular cloning: A laboratory manual (Cold Spring Harbor, NY: Cold Spring Harbor Laboratory).

Martin G.R. (1981). Isolation of a pluripotent cell line from early mouse embryos cultured in medium conditioned by teratocarcinoma stem cells. Proc. Natl. Acad. Sci. USA **78:** 1441–1445.

Miyake K., Medina K.L., Yayoshi S.I., Ono S., Hamaoka T., and Kincade P. (1990a). Monoclonal antibodies to Pgp-1/CD44 block lymphohematopoiesis in long-term bone marrow cultures. J. Exp. Med. **171:** 477–488.

Miyake K., Underhill C., Lesley J., and Kincade P. (1990b). Hyaluronate can function as a cell adhesion molecule and CD44 participates in hyaluronate recognition. J. Exp. Med. **172:** 69–75.

Mobley J.L., and Dailey M.O. (1992). Regulation of adhesion molecule expression by CD8 T cells *in vivo*. Differential regulation of $gp90^{Me114}$ (LECAM), Pgp-1, LFA-1 and VLA-4 during the differentiation of cytotoxic T lymphocytes induced by allografts. J. Immunol. **148:** 2348–2356.

Picker L.J., Nakache M., and Butcher E.C. (1989). Monoclonal antibodies to human lymphocyte homing receptors define a novel class of adhesion molecules on diverse cell types. J. Cell. Biol. **109:** 927–937.

Schmitt R.M., Bruyns E., and Snodgrass H.R. (1991). Hematopoietic development of embryonic stem cells *in vitro*: Cytokine and receptor gene expression. Genes Dev. **5:** 728–740.

Schtivelman E., and Bishop. J.M. (1991). Expression of CD44 is repressed in neuroblastoma cells. Mol. Cell. Biol. **11:** 546–5453.

Screaton G.R., Bell M.V., Jackson D.J., Cornelis F.B., Gerth U., and Bell J.I. (1992). Genomic structure of DNA encoding the lymphocyte homing receptor CD44 reveals at least 12 alternatively spliced exons. Proc. Natl. Acad. Sci. USA **89:** 12160–12164.

Slager H.G., Good M.J., Schaart G, Groenewoud J.S., and Mummery C. (1992). Organization of non-muscle myosin during early murine embryonic differentiation. Differentiation **50:** 47–56.

Stamenkovic I., Aruffo A., Amiot M., and Seed B. (1991). The hematopoietic and epithelial form of CD44 are distinct polypeptides with different adhesion potentials for hyaluronate-bearing cells. EMBO. J. **10:** 343–348.

Tabi Z., Lynch F., Ceredig R., Allan J.E., and Doherty P.C. (1988). Virus-specific memory T cells are Pgp-1+ and can be selectively activated with phorbol esters and calcium ionophore. Cell Immunol. **113:** 268–277.

Tölg C., Hofmann M., Herrlich P., Ponta H. (1993). Splicing choice from ten variants exons establishes CD44 variability. Nucleic Acid Res. **21:** 1225–1229.

Trowbridge I.F., Lesley J., Schulte R., Hyman R., and Trotter J. (1982). Biochemical characterization and tissue distribution of a polymorphic murine cell surface glycoprotein expressed on lymphoid tissues. Immunogenetics **15:** 299–312.

Underhill C. (1992). CD44: The hyaluronan receptor. J. Cell sci. **103:** 293–298.

Wiles M.V., and Keller G. (1991). Multiple hematopoietic lineages develop from embryonic stem cells in culture. Development **111:** 259–267.

Zhou D.F.H., Ding J.F., Picker L.F., Bargatze R.F., Butcher E.C., and Goeddel D.V. (1989). Molecular cloning and expression of Pgp-1– the mouse homolog of the human H-CAM (Hermes) lymphocyte homing receptor. J. Immunol. **143:** 3390–3395.

